# Variations of *BRAF* mutant allele percentage in melanomas

**DOI:** 10.1186/s12885-015-1515-3

**Published:** 2015-07-04

**Authors:** Zofia Hélias-Rodzewicz, Elisa Funck-Brentano, Laure Baudoux, Chan Kwon Jung, Ute Zimmermann, Cristi Marin, Thierry Clerici, Catherine Le Gall, Frédérique Peschaud, Valérie Taly, Philippe Saiag, Jean-François Emile

**Affiliations:** 1 EA4340, Versailles University, Boulogne-Billancourt, France; 2 Department of Pathology, Ambroise Paré Hospital, APHP, Boulogne-Billancourt, France; 3 Department of Dermatology, Ambroise Paré Hospital, APHP, Boulogne-Billancourt, France; 4 Department of Hospital Pathology, College of Medicine, The Catholic University of Korea, Seoul, Korea; 5 Department of Surgery, Ambroise Paré Hospital, APHP, Boulogne-Billancourt, France; 6 INSERM UMR-S1147, University Paris Sorbonne Cite, Paris, France

**Keywords:** Melanoma, *BRAF*, Aneuploidy, Heterozygosity

## Abstract

**Background:**

*BRAF* mutations are present in 40 % of human skin melanomas. Mutated tumors with an increased percentage of *BRAF* mutant alleles (BRAF-M%) may have a better response to RAF/MEK inhibitors. We evaluated the BRAF-M% in melanomas, and the genetic causes of its variation.

**Methods:**

BRAF-M% was quantified by pyrosequencing, real-time PCR (rtPCR) and/or picoliter-droplet PCR (dPCR). BRAF mutant expression was detected by immunohistochemistry. Chromosomal alterations were analyzed with fluorescence *in situ* hybridization (FISH), and single nucleotide polymorphism (SNP) arrays.

**Results:**

BRAF-M% quantification obtained with pyrosequencing was highly correlated (R = 0.94) with rtPCR, and with dPCR. BRAF-M% quantified from DNA and RNA were also highly correlated (R = 0.98). Among 368 samples with >80 % tumor cells, 38.6 % had a *BRAF*^*V600E*^ mutation. Only 66.2 % cases were heterozygous (BRAF-M% 30 to 60 %). Increased BRAF-M% (>60 %) was observed in 19 % of cases. FISH showed a polysomy of chromosome 7 in 13.6 %, 35.3 % and 54.5 % of *BRAF* wild-type, heterozygous and non-heterozygous *BRAF*-mutated samples, respectively (*P* < 0.005). Amplification (5.6 %) and loss (3.2 %) of *BRAF* locus were rare. By contrast, chromosome 7 was disomic in 27/27 *BRAF*-mutated nevi.

**Conclusions:**

BRAF-M% is heterogeneous and frequently increased in *BRAF*-mutant melanomas. Aneuploidy of chromosome 7 is more frequent in *BRAF* mutant melanomas, specifically in those with high BRAF-M%.

**Electronic supplementary material:**

The online version of this article (doi:10.1186/s12885-015-1515-3) contains supplementary material, which is available to authorized users.

## Background

Since the discovery of gain of function mutations in the proto-oncogenes *NRAS* [1] and *BRAF* [2], thousands of human skin melanoma samples have been analyzed, and the estimated incidence of *NRAS* and *BRAF* mutations are 18 % and 41 %, respectively [3]. These mutations are often mutually exclusive [4, 5]. The *BRAF* locus is localized on chromosome 7q, and most *BRAF* mutations involve the kinase activation loop at the p.V600 position. The most common *BRAF* mutation is a substitution of a valine to a glutamic acid (c.1799 T > A, p.V600E). *BRAF* V600E accounts for 85 % of exon 15 mutations in the most recent studies [6, 7]. Another mutation, V600K is present in 9 % of melanomas. These *BRAF* mutations constitutively activate the MAPK signaling pathway [8].

Two BRAF inhibitors, vemurafenib and dabrafenib, targeting the BRAF p.V600 mutated protein, have recently been shown to prolong the progression-free and/or the overall survival of *BRAF* V600-mutated advanced melanoma, as compared to dacarbazine [9–11]. However, both are limited by the development of acquired resistance in many patients, with a median progression-free survival (PFS) of 6.9 and 6.7 months for vemurafenib and dabrafenib, respectively [10, 12].

Mechanisms underlying acquired resistance to BRAF inhibitors have been extensively studied, and most of them involve acquired mutations within the same RAS-RAF-ERK pathway [13]. By contrast, only little data is available concerning biomarkers of good/prolonged response to BRAF inhibitors. Recently, a high ratio of mutant/wild-type alleles of *BRAF* was reported to be associated with a good response to BRAF inhibitors [14].

Like most oncogenes, somatic mutations of *BRAF* are thought to be heterozygous in tumors. Some studies reported that *BRAF* mutations are not heterozygous in some cases [15]. Additionally, in contrast to wild-type BRAF, which is only active as a dimer, products of alleles with gain of function mutations are also active as monomers [16].

We present herein a validated quantification of mutated *BRAF* in a large series of human skin melanoma samples, and demonstrate that several cases are not heterozygous. We also present the results of a genetic study on mechanisms of the *BRAF* mutant allele increase in melanoma.

## Methods

### Samples and nucleic acid extraction

All samples were obtained from the bank of biological resources of Ambroise Paré Hospital. All surgical or fine needle biopsies were performed for routine diagnosis or evaluation of disease progression. The research was performed in compliance with the ethical principles of the Helsinki Declaration (1964). In accordance with French ethics laws, all patients were informed that part of their samples could also be used for research purposes, and that they could refuse this. None of patients refused the use of samples for research. Tumor sample collection was declared to the French Ministry of Research (DC 2009-933) and CPP IDF 8 ethics committee approved the MelanCohort study (030209), which is registered with Clinicaltrials.gov (NCT00839410). Signed informed consent for translational research was obtained from patients still alive. All diagnoses were confirmed by pathology review.

For most of the nucleic acid quantification studies, the tumor DNA was extracted from formalin fixed paraffin embedded (FFPE) tissue. However, for mRNA extraction and high density SNP analysis, frozen samples were used. In all cases, a 4 micrometers-thick section was stained with hematoxylin & eosin and reviewed by a pathologist before extraction, to confirm the presence of melanoma and to select areas with the highest density of tumor cells for macrodissection. For all samples, tumors cell content was estimated in the percentage of tumor cells and the data was noted. To evaluate the accuracy of tumor cell content assessment, a series of 41 randomly selected samples was assessed by three independent pathologists.

For each sample, serial sections or punch sampling were then used for nucleic acid extraction. For DNA extraction, samples were digested by an overnight incubation in the presence of proteinase K, followed by the application of the QIAamp DNA mini kit (Qiagen, Courtaboeuf, France) as previously described [17]. The RNeasy Mini Kit (Qiagen) was applied for RNA extraction. DNA and RNA were controlled with a spectrophotometer (ND-100, Nanodrop®).

### Real-time PCR

Real-time PCR (rtPCR) was performed as previously described [18]. 1 μL of DNA brought to 25 ng/μL was applied to each reaction mixture. The amplification reaction was performed in Applied Prism 7900 HT (Thermo Fisher Scientific, Illkirch, France). Each sample was analyzed in two different reaction mixes: in the first one, all *BRAF* alleles present in the tumors were amplified; in the second one, only the mutated allele was detected by peptide nucleic acids (PNAs)-specific inhibition of wild-type (WT) allele amplification. Each PCR reaction was carried out in duplicate. The primers and probe sequences were published previously [17]. The relative quantification method was used to compare expression levels of wild type allele and *BRAF* V600E mutated allele using comparative Ct method as described by Livak *et al*. [19].

### Picoliter-droplet digital PCR

Picoliter-droplet digital PCR (dPCR) testing was performed using previously described protocols [20, 21] with the Raindrop Instrument (RainDance Technologies, Billerica, MA). Shortly, in a pre-PCR environment, 12.5 μL Taqman Genotyping Master Mix (Life Technologies, Saint Aubin, France) was mixed with the assay solution. The assay solution contained: 0.75 μL of 40 mM dNTP Mix (New England BioLabs, Evry, France), 0.5 μL of 25 mM MgCl2, 2.5 μL of 10x Droplet Stabilizer (RainDance Technologies), 1.25 μL of 20x Taqman® Assay Mix containing 8 μM of forward and reverse primers, 200 nM of 6-FAM and 200 nM of VIC Taqman® labelled-probes (Additional file 1) and target DNA template to a final reaction volume of 25 μL. A minimum of 280 ng of DNA was used in each assay. 5 millions highly monodispersed droplets were generated using the Raindrop source instrument following manufacturer’s instructions. The emulsions were submitted to thermocycling, starting with 2 min at 50 °C, 10 min at 95 °C, followed by 45 cycles of: 95 °C, 15 s and 60 °C, 1 min (using a 0.6 °C/min ramp rate). After completion, the end-point fluorescence signals from each droplet were measured using the Raindrop Sense instrument. Analyses of the data were performed using the Raindrop analyst software as previously described [20, 21]. The reference sequence was B-RAF cDNA sequence (GenBank NM_00433.4).

### Pyrosequencing

Pyrosequencing was performed as previously described [22]. It is a method of DNA sequencing based on the "sequencing by synthesis" principle. The results are displayed in the form of peaks corresponding to the detection of pyrophosphate release after nucleotides incorporation (pyrogram). Peak area is proportional to the number of individual nucleotide incorporated to the sequence; thus allowing the relative quantifications of mutated and WT alleles. RNA was transcribed to cDNA and FFPE tumor DNA concentrations were brought to 10 and 20-25 ng/μL prior to PCR amplification. Primers used for FFPE tumor DNA amplification and pyrosequencing were published previously [17]. Biotinylated amplicon was verified on agarose gel and analyzed with PyroMark 24 (Qiagen) according to manufacturer recommendations. Primers used for frozen tumor DNA/RNA amplification and pyrosequencing are presented in Additional file 1.

### FISH and Immunohistochemistry

Tissue microarray (TMA) was performed for 141 samples of melanomas (140 patients) and 42 samples of melanocytic nevi (junctional, intradermal and compound) of more than 4 mm long axis. For each tumor, three cores of 0.6 mm diameter from distinct tumor regions were spotted onto the slides.

For immunohistochemistry, the VE1 antibody was used as previously described [17, 23]. Detection of BRAF p.V600E mutated protein with VE1 has been shown to have a high sensitivity, specificity and reproducibility. Intensity of staining was evaluated by two independent observers on a semi-quantitative scale of 0–3. The VE1 antibody was scored as negative (0) when there was no staining, weak staining of single interspersed cells, or staining of monocytes/macrophages. Positive staining was scored as: weakly positive staining (1), moderately positive staining (2) and strongly positive staining (3) of melanoma cells. Cases were considered not interpretable when nuclear staining was present. Cases were scored as ambiguous if immunostaining could not be scored as positive or negative.

For FISH analysis, TMA section slides of 4 micrometers were stored at -20 °C and hybridization was performed within 2 weeks of cutting. All samples were analyzed with the RP11-121G9 BAC probe covering the *BRAF* gene. The chromosome 7 centromere probe was used as reference probe (Agilent Technologies, Les Ulis, France). A total of 53 tumor samples were also analyzed with the commercially available *BRAF* probe, SureFISH 7q34 BRAF (Agilent Technologies), together with the chromosome 7 centromere probe. For home-made probes, bacteria carrying a BAC vector were grown overnight onto solid agar medium, followed by an overnight proliferation in a LB medium. BAC DNA was extracted using NucleoBond PC 500 or NucleoBand Xtra BAC Kits (Macherey-Nagel, Hoerdt, France) as recommended by supplier. 1 μg of DNA was labeled by nick-translation reaction according to manufacturer’s instructions (Abbott Molecular Inc., Rungis, France). After overnight precipitation at -20 °C in the presence of human cot DNA, sodium acetate and ethanol, the probe was resuspended in the hybridization buffer (LSI/WCP Hybridization Buffer)(Abbott Molecular Inc). They were used at a final concentration of 40-50 ng/μL. FFPE slides were prepared for hybridization using Histology FISH accessory KIT (DAKO, Les Ulis, France). Commercial probes were applied according to the manufacturer’s recommendations (Agilent Technologies) and a co-denaturation of the probes and the tumor section were performed to create single-stranded DNA. The probes and the slides were denaturated separately when BRAF BAC probes were used together with commercial chromosome 7 centromere probe. Before an overnight incubation in a humidified chamber, a suppression of the repetitive sequences was performed for DNA BRAF BAC (45 min at 37 °C). After post-hybridization wash, and DAPI staining in the Vectashield® Mounting Media (Vector Laboratories, Les Ulis, France), fluorescence signals were analyzed using a Leica DM4000B microscope equipped with appropriate filters and a DFC300FX camera under the control of LAS V4.0 software (Leica). Two independent analyses were performed.

### SNP analysis

DNA was extracted from frozen melanoma samples and hybridized on HumanCore BeadChip (Infinium Ilumina®, Evry, France) according to the manufacturer’s instructions by IntegraGen. This array contains more than 240,000 highly-informative genome-wide tag SNP and over 20,000 high-value markers. Chromosome 13p, 14p, 15p, 21p and 22p markers are not represented in this array. Chromosome Y and X were only used to control the gender of the patient. All genome positions were based upon NCBIGRCh37/hg19 from UCSC Genome Bioinformatics. The genotyping data were normalized by the IntegraGen commercial platform and analyzed (copy number aberrations (CNA) and allele disequilibrium (AD)) using GenomeStudio software (version 1) (Illumina Inc).

### The Cancer Genome Atlas Dataset

For external validation of our results on an independent cohort, the skin melanoma dataset of The Cancer Genome Atlas (TCGA)(Provisional) was downloaded through the cBioPortal for Cancer Genomics website (http://www.cbioportal.org/ date March 13th 2015). We selected “All Complete Tumors (278)” of the patient/case set and entered the *BRAF* gene in the TCGA (Provisional) dataset. The cBioPortal website provided the data about mutation type, amino acid change and variant allele frequency. Analysis was performed as published [24].

### Statistic analyses

The correlations between the percentage of mutated *BRAF* in cDNA/gDNA and in rtPCR/pyrosequencing analysis were tested by estimating the coefficient of correlation. The chi2 test supplemented when necessary in Yates correction was used to analyze the differences between different BRAF-M% groups and chromosome 7 status. The t-test was used to compare tumors with less or more than 80 % of tumor cells. The results were considered significant when *P* < 0.05.

## Results

### Validation of *BRAF* mutated allele quantification

The percentage of *BRAF* mutant alleles (BRAF-M%) in patients with melanoma was evaluated with two quantitative methods. Both pyrosequencing and real-time PCR allowed quantification of the relative amount of c.1799 T > A substitution. However these methods are only used qualitatively in determining *BRAF* status in clinical practice. We analyzed DNA extracted from 77 FFPE melanoma samples with both methods, and found a high positive correlation (R = 0.94) of BRAF-M% (Additional file 2A). For 7 samples, BRAF-M% obtained by pyrosequencing was compared with the absolute number of both alleles assessed by picoliter-droplet digital PCR in limiting dilution conditions and obtained similar results (Additional file 3A, B). We thus decided to use pyrosequencing for the subsequent quantitative analysis of *BRAF* V600E mutation.

As quantification of genomic DNA obtained from FFPE samples may not be representative of *BRAF* mutated messenger RNA (mRNA), we extracted both mRNA and genomic DNA (gDNA) from 27 frozen melanoma samples. Each sample was then analyzed with pyrosequencing assays designed to be specific for either cDNA or gDNA. A high correlation (R = 0.98) of BRAF-M% between *BRAF* V600E cDNA and gDNA was observed (Additional file 2B). Thus, the BRAF-M% assessed in genomic DNA from FFPE samples by pyrosequencing may be considered representative of the relative quantities of mutated/WT *BRAF* in mRNA.

### Quantification of *BRAF* mutation

**Fig. 1:**
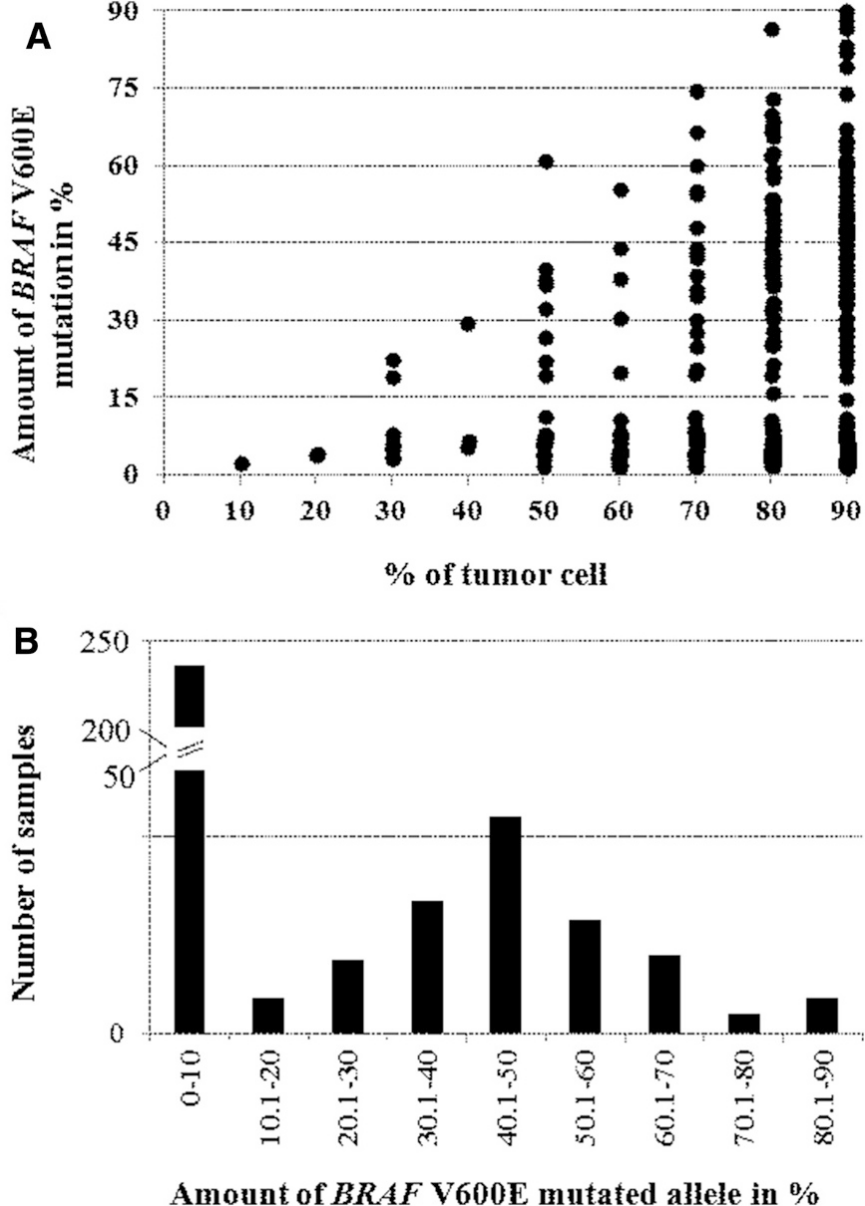
Variations in the percentage of *BRAF* V600E mutation in melanomas. **a** Scatter plot representation of the amounts of wild-type and V600E *BRAF* allele distribution in relation to the percentage of tumor cells in 475 FFPE melanoma samples. **b** Histogram representation of the percentage of *BRAF* V600E mutated allele in 368 melanomas obtained by pyrosequencing analysis. The Y axis corresponding to the number of cases is broken between 50 and 200

Pyrosequencing quantification was obtained in 475 FFPE melanoma samples from 428 patients with AJCC stage III or IV melanoma with either V600E or WT *BRAF*, after histological evaluation of the percentage of tumor cells (flow chart in Additional file 4). No discordance concerning *BRAF* mutational status was observed among the 46 patients with at least two distinct melanoma samples, and the median BRAF-M% variation was 2.5 %. As expected, according to the percentage of tumor cells in mutated *BRAF* melanomas, we observed a distinct distribution of the percentage of mutated allele (Fig. 1a). The inter-pathologist reproducibility for the evaluation of tumor cell content was substantial for the 80 % cut-off (κ = 0.79), and was only moderate for the 70 % cut-off (κ = 0.49). Thus, we decided to use the cut-off of 80 % of tumor cells. The distribution of mutated *BRAF* amounts in samples with less (n = 107) or more (n = 368) than 80 % of tumor cells were significantly different (*P* < 0.05). Therefore, we thus excluded samples with less than 80 % of melanoma cells from further analysis.

Among the 368 remaining samples, 142 were considered *BRAF* mutated (38.6 %); however BRAF-M% was highly heterogeneous (Fig. 1b), ranging from 10 % to 90 %. The majority of *BRAF* mutant cases (66.2 %, n = 94) had 30 % to 60 % of BRAF-M%, and were therefore considered heterozygous. By contrast, 48 cases were considered non-heterozygous for the *BRAF* mutation with BRAF-M% >60 % (n = 27) or <30 % (n = 21). Most *BRAF* mutated cases with BRAF-M% from 10 % to 30 % have been confirmed with another method and/or on another sample of the same patient. Immunohistochemistry with VE1 was performed on whole slides in cases with BRAF-M% <30 % when available (n = 14/21), and none of these cases contained any tumor area with loss of BRAF mutant expression.

In order to validate our results showing heterogeneous distribution of BRAF-M% in mutated melanoma tumors, we investigated the next-generation sequenced DNA mutation data of the 104 mutated skin melanomas in the TCGA database. The BRAF-M% from TCGA database ranged from 8 to 97 %, and the distribution was in keeping with our observation (Table 1).

**Table 1:** Comparison of the percentage of *BRAF* V600E mutation between our series and the TCGA database

Mutant allele frequency	Our study No. (%)	TCGA No. (%)
<30 %	21 (14.8 %)	19 (18.3 %)^a^
30-60 %	94 (66.2 %)	60 (57.7 %)^b^
<60 %	27 (19.0 %)	25 (24.0 %)
Total	142 (100.0 %)	104 (100.0 %)

^a^includes one complex mutation (P318F;V600E) ^b^includes one complex mutation (K183E;V600E)

### Genetic causes of BRAF-M% heterogeneity

**Fig. 2:**
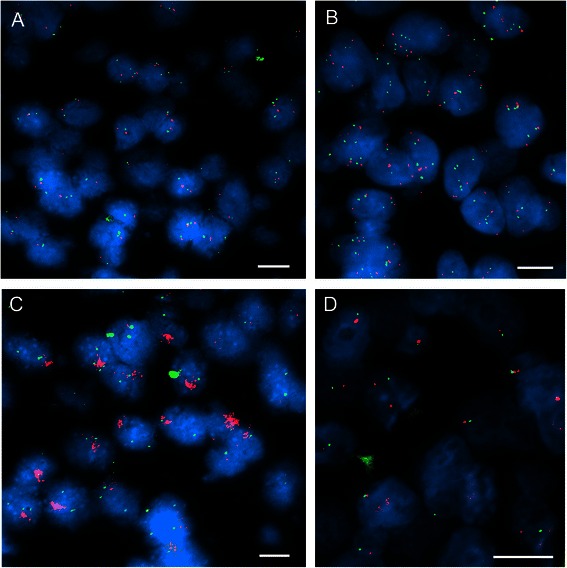
Chromosome 7 alterations in human melanomas by FISH. Representative images of FISH with *BRAF*/chromosome 7 centromere probes in melanomas with different chromosome 7 alterations. **a** no alteration (disomy) but rare cells with chromosome 7 polysomy; **b** chromosome 7 polysomy; **c** *BRAF* amplification; d) chromosome 7 monosomy. White bar = 10 μm

To elucidate mechanisms leading to BRAF-M% variation, we analyzed copy number alterations of chromosome 7 and *BRAF* locus. FISH was performed on TMA with two probes specific for the *BRAF* locus and for the chromosome 7 centromere. Among 125 samples, four types of chromosome 7 alterations were observed with *BRAF* probe RP11-121G9/centromere 7 (Fig. 2): no alteration (disomy), disomy but rare cells with polysomy, polysomy, and monosomy, which were respectively detected in 18.4 % (n = 23), 43.2 % (n = 54), 29.6 % (n = 37) and 3.2 % (n = 4) of cases. *BRAF* locus was amplified (6 to numerous *BRAF* copies) in 5.6 % of cases (n = 7).

FISH was also performed on 44 samples with another probe for the *BRAF* locus (SureFISH 7q34 *BRAF*) and similar results were obtained in all cases. To further confirm FISH results, we analyzed single nucleotide polymorphisms on chromosome 7 in 18 samples, and again all genomic results obtained were concordant with FISH (Table 2 and Additional file 5).

**Table 2:** Summary of chromosome 7/*BRAF* genetic status by SNP array and FISH analyses in melanomas

Samples	Tumor polyploidy^1^	BRAF Chromosome 7q34		FISH^2^	Amount of mutated allele by pyrosequencing	% of tumor cell	V600 status	Chromosome 7 rearrangements^3^
		Copy number	Allele disequilibrium					
B243039	Diploid	2	AB	2	2	90	WT	7pA/7qAB
B227278	Diploid	2	AB	2	2	90	WT	7pter-7q35 AB/7q35-qter A
B231355	Poly 3	2	AA	3	3	80	WT	7pter-7p21.3 AAB/7p21.3 AAB/7p21.3-p15.2AAABB/7p15.2-14.3 AAB/7q11.1-q11.2AAABB^a^/7q11.2-q11.3 AAB^a^/7q11.3-q21.11AAABB^a^/7q21.11-q31.1 AAB^a^/7q31.1-7qter AA^a^
B228256	Poly 3	3	AAB	3	3	90	WT	Non
B210964	Diploid	3	AAB	4A	3	80	WT	7pAABB/7qAAB/7qAA/7qAAB 7q complex rearrangements
B233522	Diploid	2	AB	2	4	90	WT	Non
B202779	Diploid	2	AB	NR	4	90	WT	Non
B232220	Diploid	2	AB	3	5	90	WT	Non
B236394	Diploid	2	AB	NR	5,5	90	WT	Non
B233492	Diploid	2	AB	3	35,5	90	V600E	Non
B235230	Diploid	3	AAB	3	43	80	V600E	Non
B239110	Poly 3	4	AABB	NR	45	90	V600E	Non
B230177	Diploid	2	AB	3	46	90	V600E	Non
B226709	Poly 3	4/5	AABB/AAABB^a^	4B	48	90	V600E	7pter - 7q11.2 AAB/7q11.2-q35 AAABB/q35-qter AABB
B230962	Poly 3	5	AAABB	4B	50	80	V600E	7pter-7q11.22 AABB/7q11.22-7qter AAABB
B236134	Poly 3	4/5	AABB/AAABB^a^	4A	51,5	90	V600E	7pter-7p21.1 AABB/7p21.1-p12.1 AAB/7p21.1-7q31.1 AABB/7q31.1-qter AABB/AAABB^a^
B227502	Diploid	3	AAB	NR	55,5	80	V600E	7p-7q22.3 AB/7q22.3-qter AAB
B217182	Poly 3	3	AAB	4A	61	80	V600E	Non
B230859	Poly 3	5	AAABB	3	68,5	80	V600E	7pter-p21.1 AAB/7p21.1-7p11.1 AB/7qAAABB
B231121	Poly 3	3	AAB	3	74,5	90	V600E	7pter-7q13 AB/7p13-7qter AAB
B230002	Diploid	3	AAA	4A	NA	90	V600K	7pter-7q21.1 AA/7q21.1-q34 AAA/7q34-qterAA
B223249	Diploid	4	AAAA	NI	NA	80	V600K	7pAB/7qAAA

^1^Tumor ploidy level was estimated by copy number analysis of SNP data

^2^*BRAF*/chromosome 7 centromere interpretation of FISH analysis: 2 – cells with two copies of chromosome 7; 3 – cells with two copies of chromosome 7 but rare cells showing an increased number of chromosome 7; 4 – cells with chromosome 7 gains, (A) chromosome 7 in 3 or 4 copies, (B) chromosome 7 in more than 4 copies, NI – not interpretable

^3^Chromosome 7 genetic status presents as disequilibrium of alleles A and B (SNP analysis).

NA - not available, WT – wild-type,^a^ - clonal aberration

**Fig. 3:**
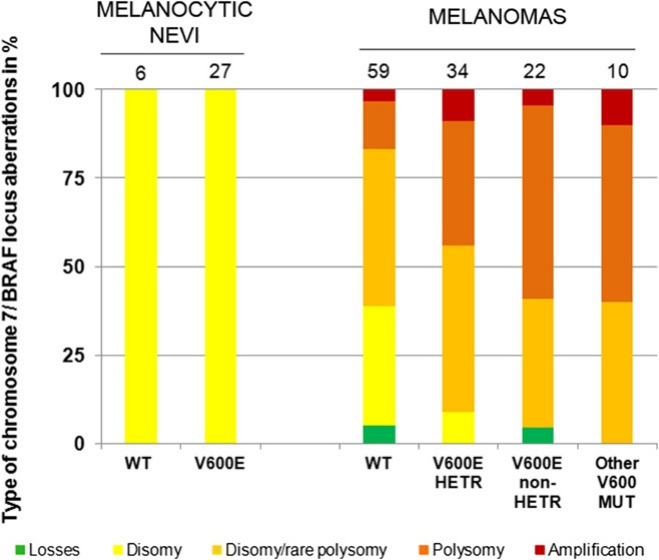
Frequency of chromosome 7 aberrations in *BRAF* mutant melanoma (n = 125) and melanocytic nevi groups (n = 33).Histogram representation of prevalence of chromosome 7 abnormalities evaluated by FISH in 115 melanomas depending on the amounts of V600E mutation, in 10 melanomas with *BRAF* exon 15 mutations different from V600E and in 33 melanocytic nevi. WT – wild-type, HETR – heterozygous, MUT – mutation

We then compared *BRAF* mutation allele quantity and chromosome 7 copy number changes (Additional file 6). Disomy of chromosome 7 - or only few cells with polysomy - were detected in 78 % (n = 46/59) of BRAF wild-type samples, but in only 47 % (n = 31/66) of *BRAF* mutants; however the difference didn’t reached statistical significance (*P* = 0.08). Quantification of BRAF-M% was not validated in samples with mutations other than V600E (n = 10/66). Correlation with FISH showed a polysomy of chromosome 7 in 13.6 % (n = 8/59), 35.3 % (n = 12/34) and 54.5 % (n = 12/22) of wild-type, heterozygous and non-heterozygous samples, respectively (*P* < 0.05) (Fig. 3). Furthermore, none of the 22 non-heterozygous cases, versus 33.9 % (n = 20/59) of the *BRAF* WT group, were diploid (*P* < 0.05). Among the 22 non-heterozygous samples, six had low BRAF-M% (Additional file 6). Six of the 10 samples with other *BRAF* mutations had a polysomy of chromosome 7.

### *BRAF* copy number and BRAF expression

We also investigated the VE1 data for 114 samples from TMA slides. Both VE1 and FISH data were available for 45 *BRAF* V600E mutated melanomas. The VE1 staining were stronger in mutated cases showing hyperploidy or amplification for chromosome 7 (74 %, 17/23) than in tumors with only one chromosome 7 or with no (disomy) or minor (rare cells with polysomy) alterations (32 %, 7/22) (*P* < 0.05). Interestingly, the VE1 staining was stronger in melanomas with BRAF-M% >60 % than in those <60 %, but this was not statistically significant.

### *BRAF* and chromosome 7 statuses in melanocytic nevi

The frequent chromosome 7 aneuploidy may be the cause or the consequence of *BRAF* mutations in melanomas. Alternatively, it may occur in melanocyte proliferations regardless of *BRAF* mutations. To address this question we analyzed 42 dermic and/or junctional melanocytic nevi. BRAF p.V600E mutation were detected by immunohistochemistry with VE1 on tissue arrays in 78.6 % (n = 33) of nevi. *In situ* hybridization with probes corresponding to the *BRAF* locus and the chromosome 7 centromere was interpretable in 27 of these 33 BRAF mutant benign tumors (81.2 %), and chromosome 7 was diploid in all cases (Fig. 3).

## Discussion

The primary objective of this study was to assess the percentage of *BRAF* mutated allele (BRAF-M%) in a large series of human melanoma samples. For this purpose, we first validated quantification by pyrosequencing by comparing it with 2 other methods: real-time PCR and picoliter-droplet digital PCR. We also demonstrated a close correlation of BRAF-M% quantifications in genomic DNA and messenger RNA. The best inter-pathologist reproducibility of the estimation of the percentage of tumor cells in mutated melanoma was obtained with a cut-off at 80 %. Furthermore, the mean BRAF-M% was significantly lower for cases with <80 % of tumor cells, probably due to the presence of the wild type non-tumor cells. We therefore limited the analysis to samples containing at least 80 % of tumor cells.

Using this validated quantitative method, we analyzed BRAF-M% in a series of 368 melanoma samples and found that it was very heterogeneous. We then investigated the genetic cause of this high heterogeneity by FISH and high density SNP array, and demonstrated that chromosome 7 aneuploidy was the main mechanism of unbalanced *BRAF* allelic ratio. Finally we showed that, as opposed to melanomas, benign melanocytic tumors with *BRAF* mutations did not have any chromosome 7 instability.

Although oncogenic mutations are expected to occur in only one of the two parental alleles, we found that only two thirds of *BRAF* mutated melanomas were heterozygous. Decreased (<30 %) and increased (>60 %) levels of V600E mutations were detected in 14.8 % and 19 % of mutated *BRAF* p.V600E melanomas, respectively. Unbalanced *BRAF* mutations have been previously reported to be frequent in smaller series of melanoma [14, 16, 25]; however quantitative methods were not clearly validated, and correlation with mRNA levels was not established. Our results were further confirmed by analysis of the TCGA database. These results raise important questions concerning BRAF targeted therapies. Indeed, ATP-competitive RAF inhibitors have different effects in cells expressing wild-type or mutant BRAF forms, with up- or down-regulation of the ERK signaling pathway in certain *in vitro* conditions [26]. Furthermore, activation of the wild-type form depends on dimerization, while monomeric mutant forms have been shown to be active [16]. As BRAF-M% appears to be highly heterogeneous in melanomas, one could expect differences in response to ATP-competitive RAF inhibitors between tumors. Data from a recent clinical study support this hypothesis, showing that the *BRAF* V600 mutation level was significantly associated with a better response rate to vemurafenib during the first 10 months of treatment [14]. In our series, two patients with increased *BRAF* V600E levels (86.6 % and 86.7 %) had a prolonged disease-free survival during respectively 25 and 39 months of BRAF p.V600 inhibitors therapy. These results emphasize the appeal of mutant *BRAF* quantification assessment prior to BRAF inhibitor treatment, to correlate with clinical response rate and survival. In clinical practice, the quantification of BRAF mutations deserves to be considered as a standard item in reporting the mutational status of BRAF. Furthermore, *in vitro* screening of new drugs should include cell lines with different BRAF mutant levels, thus more closely representative of BRAF-M% in patients’ melanomas. Of note, an *in vitro* study showed that 4/4 melanoma cell lines with homozygous *BRAF*^*V600E*^ mutations were sensible to vemurafenib, while 3/6 with heterozygous *BRAF*^*V600E*^ mutations were resistant [27].

Our initial hypothesis was that amplification of the BRAF locus is responsible for high BRAF-M%. However, *BRAF* amplification was observed in only 5.6 % of samples (n = 7), including 5 *BRAF* mutated cases. By contrast, we detected a polysomy of chromosome 7 in the majority of cases with high BRAF-M%. A second FISH analysis with another probe specific for the *BRAF* locus and SNP analysis on chromosome 7 confirmed the quality of our FISH data.

The low BRAF-M% may be related to the presence of non-tumor cells. Indeed, we observed a relationship between percentage of tumor cells and BRAF-M%. However cases with low BRAF-M% were also detected when including only samples with >80 % of tumor cells. Tumor heterogeneity of *BRAF* mutated melanomas, including some areas without *BRAF* mutations, have been reported by a few groups [28, 29], and could also be responsible for low BRAF-M%. Therefore, we performed an *in situ* analysis of cases with <30 % of mutant allele by immunohistochemistry with the BRAF p.V600E-specific VE1 antibody on whole slide sections. In all cases available for analysis, no negative areas were detected. Thus the main cause of low BRAF-M% is probably similar to high BRAF-M%. Unfortunately, we cannot confirm this in the present study, because only 6 cases were analyzed by FISH. Interestingly, *BRAF* mutated tumors with numerous copies of chromosome 7 displayed stronger VE1 staining, suggesting a higher expression of the *BRAF* mutant allele.

We report herein that only 18.4 % of melanomas had no alterations of chromosome 7. Other groups have already shown that chromosome instability is not restricted to chromosome 7. Indeed DNA copy number alterations were widely studied in both primary melanomas [30, 31] and melanoma cell lines [31–34] and frequent gains of 6p, 7, 8, 17q and 20q and losses of 9p, 10, 21q were reported. However, these studies did not quantify the amounts of the mutated BRAF allele. Two groups correlated chromosome 7 copy numbers with BRAF mutational status [15, 35]; however BRAF-M% was evaluated on sequence electropherogram peaks. Willmore-Payne et al. detected seven cases with chromosome 7 polysomy and two with *BRAF* amplification [15]. The percentage of *BRAF*-mutated and WT alleles were compared with 100 K SNP chip data for chromosome 7 by Spittle and colleagues [25]. However, this analysis, carried out in eight melanoma cell lines, showed the preferential amplification of mutant *BRAF* as a mechanism of an increased ratio of mutant/WT *BRAF*.

We detected the *BRAF* p.V600E mutation in 78.6 % of dermal and/or junctional melanocytic naevi. These results were obtained through immunohistochemistry with the VE1 antibody, whose specificity, sensitivity and reproducibility were demonstrated in melanomas [17, 22, 36]. A similar frequency of *BRAF* mutations in the same types of nevi has already been reported [37]. We then analyzed chromosome 7 aneuploidy in *BRAF* mutated nevi with FISH of chromosome 7. As opposed to the results obtained in melanoma, no alterations were detected in the 27 cases of nevi available, thus excluding a causal link between *BRAF* mutations and chromosomal instability in melanocytic tumors. These results are in keeping with previously published data [38]. *BRAF* was previously proposed to be the driver of copy number increase in melanoma [35]. The present data do not support this hypothesis. However, association of chromosome 7 aneuploidy with malignancy was significant in *BRAF* mutated melanocytic tumors (35/66 versus 0/27, *P* < 0.05). Our results suggest that, among the high number of genetic alterations present in melanomas, a frequent oncogenic pathway is characterized by an early gain of function mutation in *BRAF* and a late transforming mutation in another gene responsible for chromosomal instability. However, this has to be confirmed in cell models.

## Conclusions

BRAF inhibitors are widely used to treat patients with *BRAF* mutated melanoma, but most metastatic patients with initial tumor response develop acquired resistance with a median PFS of less than 7 months [10, 12]. Biomarkers of long-term response are still missing; however a high BRAF-M% was recently reported to be correlated with a prolonged response. We show here that only two thirds of *BRAF* V600E melanomas have a heterozygous mutation. Thus quantitative, rather than qualitative, evaluation of *BRAF* mutation deserves to be considered as a standard item in reporting the mutational status of melanoma. Prospective clinical studies are necessary to determine the BRAF-M% prognostic impact in term of response rate or prolonged response to BRAF inhibitors, and to confirm that BRAF-M% could be used as a biomarker of long-term response in clinical practice.

## Additional files

Additional file 1:
**Oligonucleotides used for** ***BRAF*** **gDNA/cDNA analysis and** ***BRAF*** **dPCR.**

Additional file 2: **Correlation analysis of BRAF V600E mutated allele frequency.** *A*) in 77 FFPE melanoma samples by pyrosequencing and rtPCR. B) in gDNA and cDNA in 27 frozen melanoma samples.
Additional file 3: **Picoliter-droplet digital PCR (dPCR) analysis for*****BRAF*****V600E in melanomas.** A) Representative results of dPCR analysis for *BRAF* V600E on DNA obtained from 2 melanomas with different percentage of mutated allele evaluated by pyrosequencing. On the x and y axis are reported the fluorescence intensity of droplets positive for *BRAF* V600E and *BRAF* wild-type, respectively. The percentage of *BRAF* V600E, *BRAF* wild-type and empty droplets are reported in images. B) Comparison of *BRAF* mutated allele frequency detected by pyrosequencing and dPCR for 7 melanoma samples.
Additional file 4:
**Workflow for melanoma and melanocytic nevi samples regarding the type of molecular analysis carried out.**

Additional file 5: **Chromosome 7 alterations by Illumina BeadChip HumanCore SNP array.** Examples of chromosome 7 alterations in 5 *BRAF* V600E mutated and in one *BRAF* wild-type melanoma (C). In each case, logR_2_ ratios for the SNP and B allele frequency are plotted on the X axis above the chromosome ideogram. For logR_2_ ratios, values centered on 0 indicate diploid copy number. Values under and above 0 indicate losses and gains respectively. For B allele frequency, values differ from around 0.5 indicated LOH.
Additional file 6: **Summary of pyrosequencing and FISH results.** BRAF-M% and FISH results obtained for 125 melanomas analyzed by FISH with BRAF BAC/chromosome 7 centromere probes.

## Abbreviations

BAC: Bacterial artificial chromosome
BRAF-M%: Percentage of *BRAF* mutant alleles
dPCR: Picoliter droplet PCR
FFPE: Formalin fixed paraffin embedded
FISH: Fluorescence *in situ* hybridization
PFS: Progression-free survival
rtPCR: real-time PCR
SNP: Single nucleotide polymorphism
TMA: Tissue microarray
WT: Wild type
