# Supplementary material for: Variations of BRAF mutant allele percentage in melanomas
Source: BMC Cancer. 2015 Jul 4;15:497. doi: 10.1186/s12885-015-1515-3 (PMC4491198; doi:10.1186/s12885-015-1515-3)
Supplement: Additional file 1: — Oligonucleotides used for BRAF gDNA/cDNA analysis and BRAF dPCR. [file 12885_2015_1515_MOESM1_ESM.pdf]

PCR and pyrosequencing primers specific for *BRAF* V600 gDNA and cDNA

gDNA	Sequences
**Forward Primer**	TTCATGAAGACCTCACAGTAAAAA
**Reverse Primer**	TTCTAGTAACTCAGCAGCATCTC
**Pyrosequencing Primer**	ATGGGACCCACTCCAT
**cDNA**	**Sequences**
**Forward Primer**	TTCATGAAGACCTCACAGTAAAAA
**Reverse Primer**	GACTGAAAGCTGTATGGATTTT
**Pyrosequencing Primer**	ATGGGACCCACTCCAT

***BRAF* V600 Taqman primers and probes for picoliter-droplet digital PCR**

	Sequences
**BRAF F**	CTACTGTTTTCCTTTACTTACTACACCTCAGA
**BRAF R**	ATCCAGACAACTGTTCAAACTGATG
**BRAF V600 WT**	VIC-CTAGCTACAGTGAAATC
**BRAF V600 MUT**	6FAM-TAGCTACAGAGAAATC
