# Supplementary figures and images for: Variations of BRAF mutant allele percentage in melanomas
Source: BMC Cancer. 2015 Jul 4;15:497. doi: 10.1186/s12885-015-1515-3 (PMC4491198; doi:10.1186/s12885-015-1515-3)

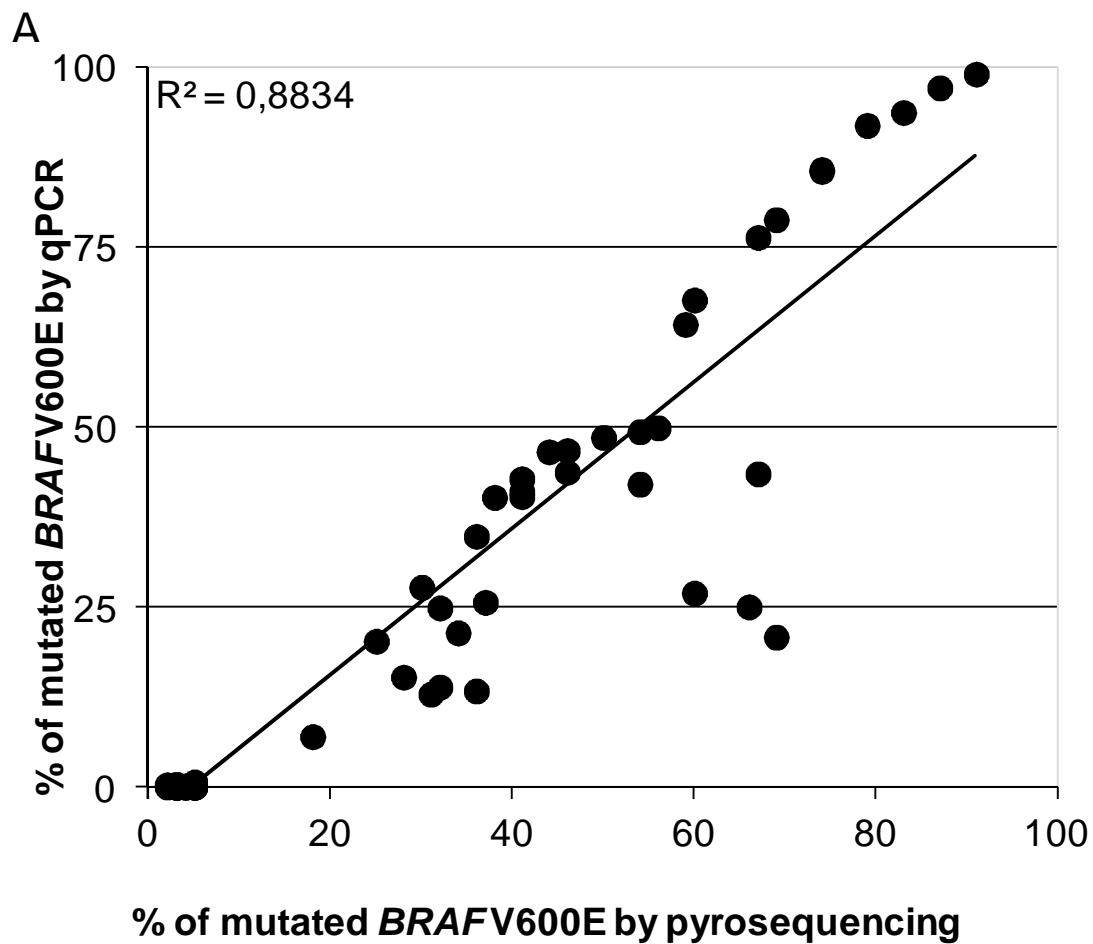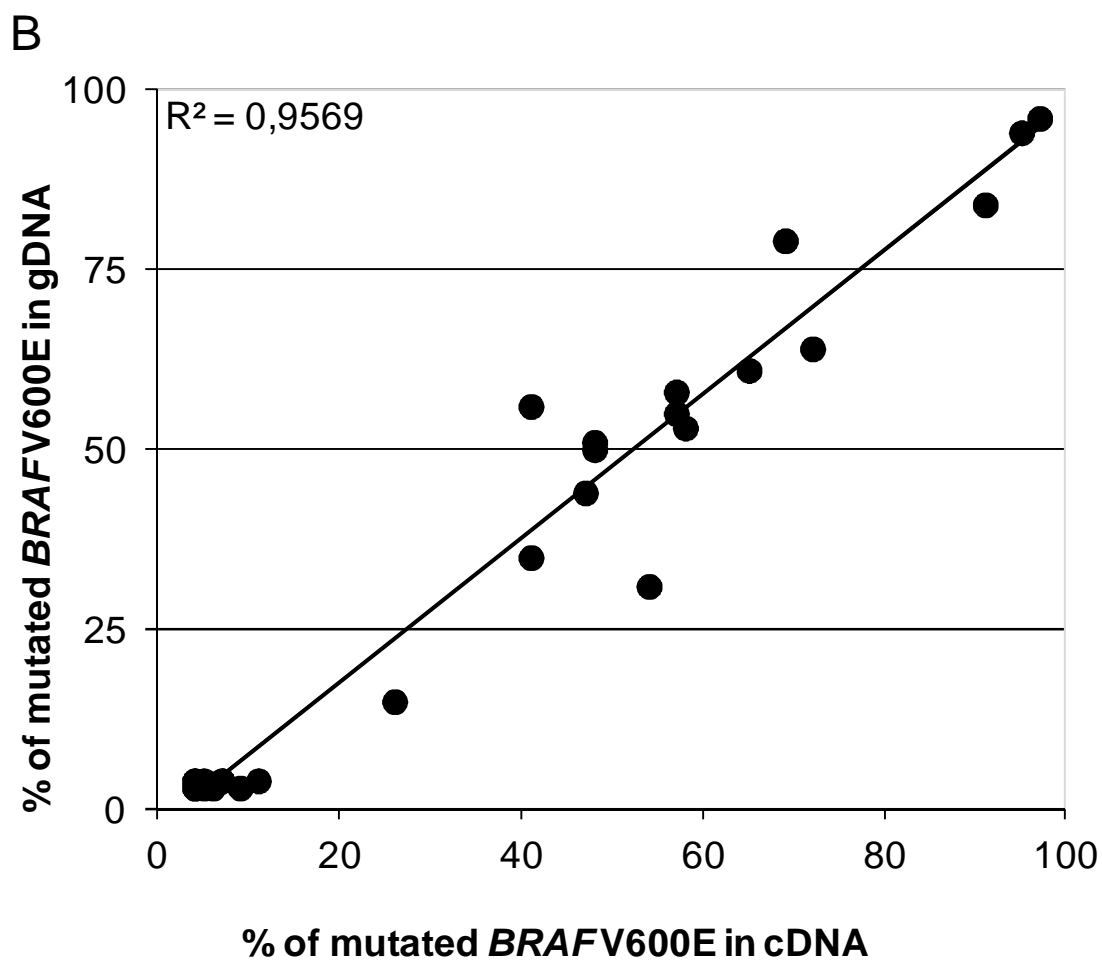

Supplement: Additional file 2: — Correlation analysis of BRAF V600E mutated allele frequency. A) in 77 FFPE melanoma samples by pyrosequencing and rtPCR. B) in gDNA and cDNA in 27 frozen melanoma samples. [file 12885_2015_1515_MOESM2_ESM.pdf]

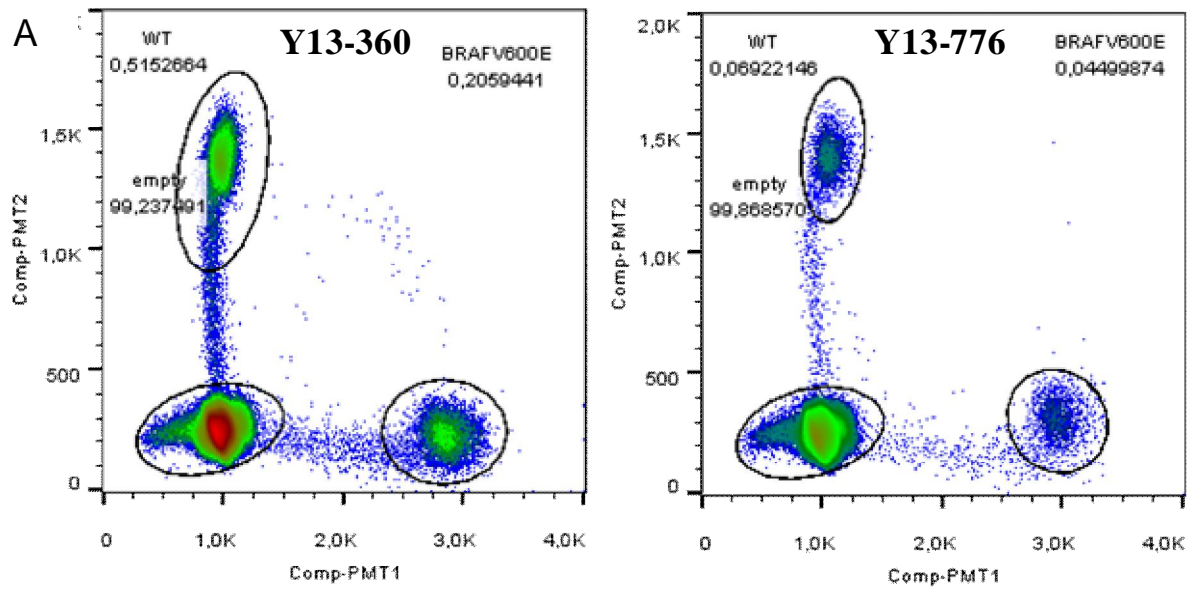

**B**

| Samples | No. of WT/V600E<br>genomes by dPCR | % of mutated BRAF by |                |
|---------|------------------------------------|----------------------|----------------|
|         |                                    | dPCR                 | pyrosequencing |
| Y13-468 | 5561/1219                          | 18.0                 | 21.5           |
| Y13-360 | 21468/8477                         | 28.3                 | 30.5           |
| Y13-776 | 2892/1872                          | 39.3                 | 42             |
| Y13-992 | 3701/3790                          | 50.6                 | 49.5           |
| Y13-332 | 90/133                             | 59.6                 | 57.5           |
| Y13-158 | 16486/28857                        | 63.6                 | 65             |
| Y13-443 | 1134/3306                          | 74.4                 | 73             |

Supplement: Additional file 3: — Picoliter-droplet digital PCR (dPCR) analysis forBRAFV600E in melanomas. A) Representative results of dPCR analysis for BRAF V600E on DNA obtained from 2 melanomas with different percentage of mutated allele evaluated by pyrosequencing. On the x and y axis are reported the fluorescence intensity of droplets positive for BRAF V600E and BRAF wild-type, respectively. The percentage of BRAF V600E, BRAF wild-type and empty droplets are reported in images. B) Comparison of BRAF mutated allele frequency detected by pyrosequencing and dPCR for 7 melanoma samples. [file 12885_2015_1515_MOESM3_ESM.pdf]

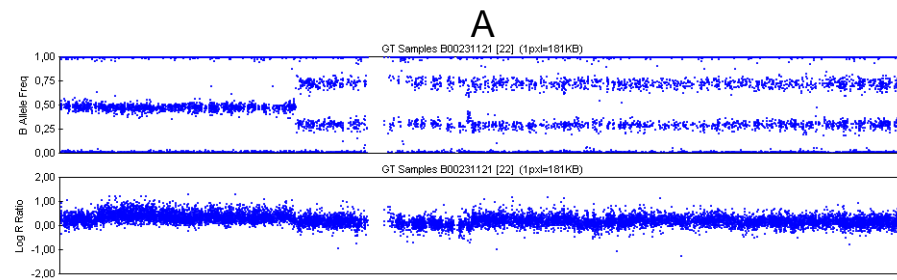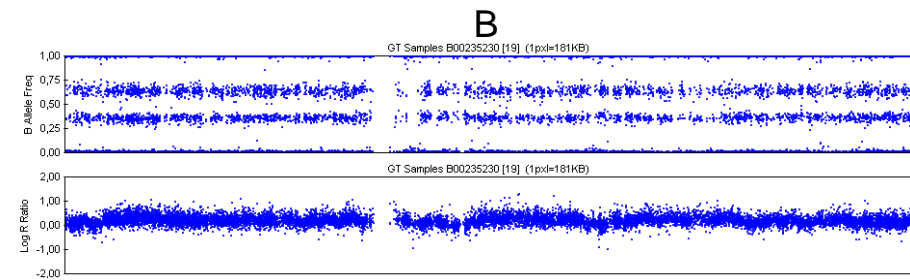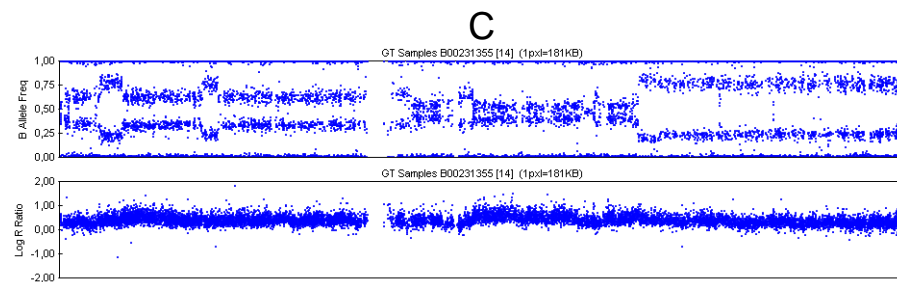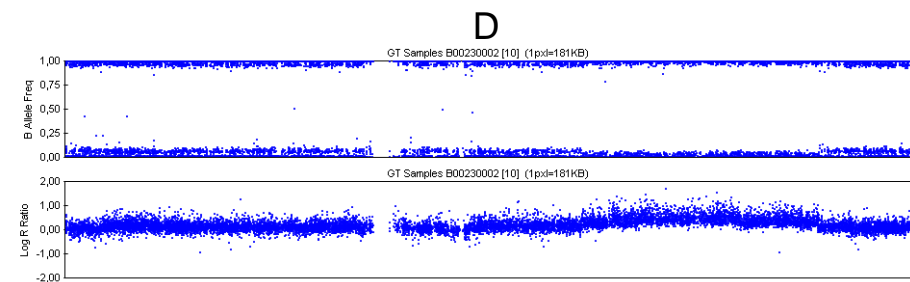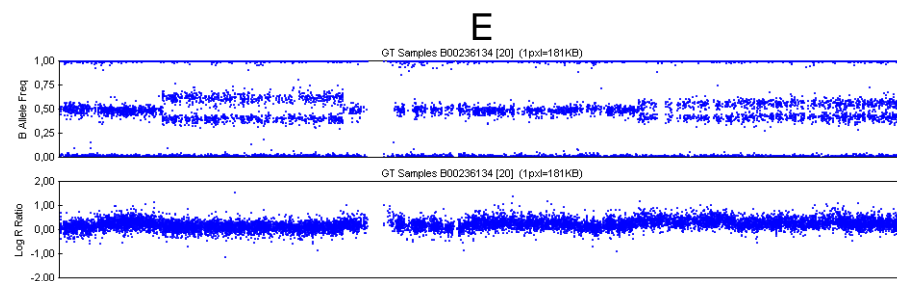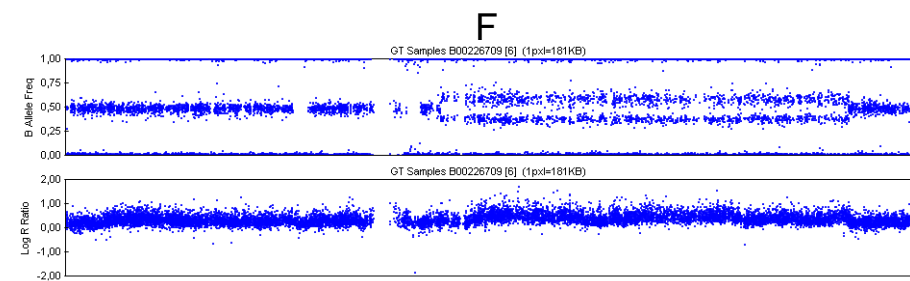

Supplement: Additional file 5: — Chromosome 7 alterations by Illumina BeadChip HumanCore SNP array. Examples of chromosome 7 alterations in 5 BRAF V600E mutated and in one BRAF wild-type melanoma (C). In each case, logR2 ratios for the SNP and B allele frequency are plotted on the X axis above the chromosome ideogram. For logR2 ratios, values centered on 0 indicate diploid copy number. Values under and above 0 indicate losses and gains respectively. For B allele frequency, values differ from around 0.5 indicated LOH. [file 12885_2015_1515_MOESM5_ESM.pdf]
