# Supplementary material for: Variations of BRAF mutant allele percentage in melanomas
Source: BMC Cancer. 2015 Jul 4;15:497. doi: 10.1186/s12885-015-1515-3 (PMC4491198; doi:10.1186/s12885-015-1515-3)
Supplement: Additional file 4: — Workflow for melanoma and melanocytic nevi samples regarding the type of molecular analysis carried out. [file 12885_2015_1515_MOESM4_ESM.pdf]

MELANOMA SAMPLES

Percentage of *BRAF* V600E mutation obtained by pyrosequencing

475 samples from 428 patients. All consecutive samples with stage III or IV melanomas received in Ambroise Paré hospital pathology department from March 2010 to May 2014

→ 368 samples with at least 80% of tumor cells of whom 142 with *BRAF* V600E

Fluorescence *in situ* hybridization (FISH) with BRAF (RP11-129G9 BAC)/chromosome 7 centromere probes

141 melanoma samples available on TMA (140 patients)
16 not interpretable because of lack of tumor on TMA sections, high lymphocytic infiltration or high background fluorescent signals.

→125 samples with interpretable results

59 *BRAF* WT

56 *BRAF* V600E

10 with another *BRAF* exon 15 mutations (V600K n=7, K601E n=2, 600-601delinsE n=1).

→44 samples analyzed with another BRAF probe (SureFISH 7q34 BRAF)

Single nucleotide polymorphism (SNP) array analysis

18 frozen samples available

→ 17 samples with FISH results

→ 1 sample with non interpretable FISH result

MELANOCYTIC NEVI (dermic and/or junctional)

FISH with BRAF (RP11-129G9 BAC)/chromosome 7 centromere probes and immunohistochemitry (IHC) with VE1 antibody

→ 42 samples from 42 patients

IHC

→ 42 samples interpretable

9 BRAF p.V600E negative

33 BRAF p.V600E positive

FISH analysis

→33 samples with interpretable FSIH results

6 BRAF p.V600E negative

27 BRAF p.V600E positive

9 FISH not interpretable because of lack of tumor on TMA sections
