# Supplementary material for: Variations of BRAF mutant allele percentage in melanomas
Source: BMC Cancer. 2015 Jul 4;15:497. doi: 10.1186/s12885-015-1515-3 (PMC4491198; doi:10.1186/s12885-015-1515-3)
Supplement: Additional file 6: — Summary of pyrosequencing and FISH results. BRAF-M% and FISH results obtained for 125 melanomas analyzed by FISH with BRAF BAC/chromosome 7 centromere probes. [file 12885_2015_1515_MOESM6_ESM.pdf]

BRAF WT group No=59

Samples	FISH results	BRAF-M%
Y10.1644	2	4
Y10.1645	2	4
Y10.1759	2	3.3
Y10.1766	2	4
Y10.1768	2	6
Y10.1771	2	7
Y10.1779	2	3
Y10.1788	2	3
Y10.1802	2	3
Y10.1836	2	3
Y10.880	2	3
Y11.190	2	3
Y11.991	2	2
Y12.138	2	5.5
Y12.198	2	4.5
Y12.205	2	2.5
Y12.300	2	4
Y12.514	2	4.5
Y12.578	2	5
Y12.658	2	2
Y10.1471	3	3
Y10.1526	3	3
Y10.1589	3	3.5
Y10.1646	3	3
Y10.1647	3	3
Y10.1772	3	5
Y10.1777	3	5
Y10.1778	3	3.5
Y10.1780	3	4
Y10.1787	3	4
Y10.1806	3	2.5
Y10.2113	3	3
Y11.189	3	3
Y11.5	3	3
Y11.7	3	3
Y11.826	3	6
Y11.996	3	3
Y12.255	3	6
Y12.278	3	5
Y12.316	3	3
Y12.36	3	4
Y12.461	3	5
Y12.616	3	3.5

FISH

2 = disomy of chromosome 7

3 = disomy of chromosome 7 but rare cells with chromosome 7 polysomy

4 = polysomy of chromosome 7

5 = BRAF amplification or gain

6= monosomy of chromosome 7

Samples	FISH results	BRAF-M%
Y12.627	3	4
Y12.878	3	10
Y12.882	3	5.5
Y12.39	5	3.5
Y10.1822	5	5
Y10.1470	6	2
Y10.1752	6	4
Y13.50	6	5.5
Y12.1041	4A	2
Y10.1898	4A	3
Y12.636	4A	3.5
Y12.657	4A	4.5
Y10.1760	4A	10
Y12.661	4B	3.5
Y10.1476	4B	4
Y10.1791	4B	4.5

BRAF V600E Heterozygous group No=34

Samples	FISH results	BRAF-M%
Y10.1764	2	41
Y12.435	2	45.5
Y12.943	2	41
Y10.1473	3	39
Y10.1651	3	36.5
Y10.1751	3	35
Y10.1753	3	54
Y10.1767	3	41
Y10.1810	3	38.5
Y10.1830	3	40.5
Y10.2001	3	35
Y11.200	3	47
Y11.6	3	32
Y12.1018	3	46
Y12.301	3	34.3
Y12.374	3	46
Y12.409	3	30.25
Y12.610	3	46
Y12.653	3	35.5
Y12.303	5	50
Y12.377	5	54
Y12.667	5	30.5
Y12.717	4A	36.5
Y12.1001	4A	51.5
Y11.13	4A	48
Y10.2002	4A	35
Y10.1818	4A	59.5
Y10.1469	4A	42
Y10.1525	4B	51.5
Y10.1650	4B	56.5
Y10.1762	4B	38
Y10.1794	4B	48
Y12.1006	4B	48
Y12.257	4B	58.5

BRAF V600E Non-Heterozygous group No=22

Samples	FISH results	BRAF-M%
Y10.1668	3	79.5
Y10.1776	3	19
Y10.1835	3	68
Y11.183	3	17
Y11.9	3	74
Y12.240	3	68.5
Y12.260	3	74.5
Y12.337	3	25.5
Y11.25	5	83
Y10.1652	6	26.5
Y12.487	4A	90
Y11.919	4A	80
Y10.2082	4A	88
Y10.1819	4A	61
Y10.1733	4A	87
Y10.1127	4A	61
Y10.1761	4B	25.5
Y10.1790	4B	67
Y10.1808	4B	28.5
Y10.1811	4B	61.5
Y12.434	4B	85
Y13.855	4B	62.5

Other *BRAF* V600 mutations group No=10

Samples	FISH results	Type of Mutation
Y10.848	3	p.V600K
Y11.701	3	p.K601E
Y12.743	3	p.V600K
Y12.781	3	p.V600K
Y12.302	5	p.V600K
Y10.1472	4A	p.V600-K601 delins E
Y10.1997	4A	p.V600K
Y10.1831	4B	p.V600K
Y11.515	4B	p.K601E
Y12.230	4B	p.V600K
